# Socioeconomic position, bronchiolitis and asthma in children: counterfactual disparity measures from a national birth cohort study

**DOI:** 10.1093/ije/dyac193

**Published:** 2022-09-30

**Authors:** Kate M Lewis, Bianca L De Stavola, Steve Cunningham, Pia Hardelid

**Affiliations:** Population, Policy and Practice Research and Teaching Department, University College London Great Ormond Street Institute of Child Health, London, UK; Population, Policy and Practice Research and Teaching Department, University College London Great Ormond Street Institute of Child Health, London, UK; Department of Child Life and Health, Centre for Inflammation Research, University of Edinburgh, Edinburgh, UK; Population, Policy and Practice Research and Teaching Department, University College London Great Ormond Street Institute of Child Health, London, UK

**Keywords:** Asthma, bronchiolitis, socioeconomic factors, longitudinal studies, inverse probability-weighting, potential outcomes, observational studies

## Abstract

**Background:**

The debated link between severe respiratory syncytial virus (RSV) infection in early life and asthma has yet to be investigated within a social inequity lens. We estimated the magnitude of socioeconomic disparity in childhood asthma which would remain if no child were admitted to hospital for bronchiolitis, commonly due to RSV, during infancy.

**Methods:**

The cohort, constructed from national administrative health datasets, comprised 83853 children born in Scotland between 1 January 2007 and 31 June 2008. Scottish Index for Multiple Deprivation (SIMD) was used to capture socioeconomic position. Emergency admissions for bronchiolitis before age 1 year were identified from hospital records. Yearly indicators of asthma/wheeze from ages 2 to 9 years were created using dispensing data and hospital admission records.

**Results:**

Using latent class growth analysis, we identified four trajectories of asthma/wheeze: early-transient (2.2% of the cohort), early-persistent (2.0%), intermediate-onset (1.8%) and no asthma/wheeze (94.0%). The estimated marginal risks of chronic asthma (combining early-persistent and intermediate-onset groups) varied by SIMD, with risk differences for the medium and high deprivation groups, relative to the low deprivation group, of 7.0% (95% confidence interval: 3.7–10.3) and 13.0% (9.6–16.4), respectively. Using counterfactual disparity measures, we estimated that the elimination of bronchiolitis requiring hospital admission could reduce these risk differences by 21.2% (4.9–37.5) and 17.9% (10.4–25.4), respectively.

**Conclusions:**

The majority of disparity in chronic asthma prevalence by deprivation level remains unexplained. Our paper offers a guide to using causal inference methods to study other plausible pathways to inequities in asthma using complex, linked administrative data.

Key Messages

- We identified four trajectories of asthma/wheeze symptoms among children aged 2 to 9 years born in Scotland: early-transient (2.2% of the cohort), early-persistent (2.0%), intermediate-onset (1.8%) and no asthma/wheeze (94.0%).
- Children born into areas with high levels of socioeconomic deprivation were more likely to experience trajectories with asthma/wheeze symptoms compared with other children.
- Using counterfactual disparity measures, we estimated that if bronchiolitis hospital admissions in infancy were eliminated, about one-fifth of the difference in the risk of chronic asthma between children from different socioeconomic groups in Scotland would be removed.
- Our paper offers a guide to the use of causal inference methods to study other plausible pathways to inequities in asthma using complex, linked administrative data.

## Introduction

Asthma is the most common chronic respiratory condition in children worldwide.^1^ In 2018, an estimated 8% of children younger than 15 years in Scotland had a current doctor diagnosis of asthma.^2^ Severe symptoms of respiratory syncytial virus (RSV) infection in infancy, including hospital admissions due to bronchiolitis, have been frequently associated with an increased risk of wheeze and asthma in later childhood,^3^ offering a possible pathway for early intervention. However, it remains unclear whether this association represents a true causal mechanism (i.e. early infection impairs pulmonary function, thereby directly influencing the development of asthma) or it is due to other shared influences, such as environmental exposures and/or genetic disposition to respiratory ill health.^3–5^ The research to date has not looked at this question within a social inequity lens, despite socioeconomic deprivation being a prevailing facet of both conditions.^6^^,^^7^ Identifying the extent to which severe bronchiolitis (i.e. that which requires hospital admission) may mediate the pathway between socioeconomic deprivation and asthma will offer guidance on the planning and potential impact of future preventive measures, including RSV vaccines and extended half-life monoclonal antibodies, which will become available within the next 5 years.^8^^,^^9^

One challenge for studies of the link between RSV infection and asthma is that ‘asthma’ is not one condition but a heterogeneous group of respiratory disorders with multiple aetiologies.^3^ In the early years in particular, recurrent symptoms of wheeze are common and are not necessarily an indicator of chronic asthma.^10^ To better investigate the heterogeneous entities (and aetiologies) that likely comprise asthma,^11^ statistical models to identify typical subgroups of asthma symptoms have been applied to longitudinal cohort studies.^12–14^ However, a 2020 systematic review and meta-analysis of childhood wheeze trajectory-specific risk factors identified high risk of information bias in all of the 13 included cohort studies.^14^ Self-reports, rather than clinical validation of symptoms, limited sample sizes, and loss to follow-up are noted problems that lead to low power to detect associations with risk factors. Overcoming many of these previous shortcomings, Sbihi *et al*. used administrative health data to identify different asthma trajectories in a sample of more than 65 000 children in British Columbia.^15^ Results of Sbihi *et al*.’s study demonstrate the unique opportunity administrative data offer to study asthma trajectories, overcoming logistic and financial constraints and self-report biases inherent in purposefully designed longitudinal cohort studies based on questionnaires.

We aimed to estimate how prevention of severe infant bronchiolitis might reduce the socioeconomic patterning of wheeze/asthma trajectories through childhood. First, we modelled typical trajectories of asthma among children from age 2 to 9 years, inclusive. Second, having observed socioeconomic disparities in prevalence of these trajectories, we established the extent to which these disparities would remain if hospital admissions for bronchiolitis during infancy could be prevented. We used national, linked administrative data from children born in Scotland to meet our aims.

## Methods

### Data sources

Several Scotland-wide administrative databases, provided by Public Health Scotland (PHS), comprised the longitudinal dataset for this study. National Records of Scotland birth registrations (the spine of the cohort) were supplemented with the Scottish Birth Record, an electronic record of neonatal care, and the mother’s delivery record (Scottish Morbidity Record 02; SMR02).^16–18^ Subsequent hospital admissions by cohort members were retrieved from SMR01, a dataset of all inpatients and day cases discharged from hospitals in Scotland.^18^ Information about deaths was added from National Records of Scotland death registrations.^19^ Data were also extracted from the Scottish national Prescribing Information System, which contains information on medicines dispensed in a community setting.^20^

PHS linked records belonging to the same child across databases using their unique Community Health Index (CHI) number.^21^ The CHI database includes information on deregistrations from Scottish general practices, which was used to estimate emigration.^22^ We received these datasets from PHS with a unique pseudo-anonymized identifier for each child and mother, and with patients names, CHI numbers and full addresses removed. Child and maternal datasets were linked by PHS using deterministic and probabilistic methods described elsewhere.^23^

### Study population

The initial study population included all live births between 1 January 2007 and 31 June 2008 in Scotland. Follow-up began at birth and continued until the 10th birthday. We excluded children who died or emigrated out of Scotland by age 2, to ensure that all children in the cohort could have had a recording of asthma/wheeze on at least one time point. Children born to non-resident mothers (as recorded on birth registrations) were excluded, to prevent potential systematic loss to follow-up. One child from each non-singleton birth was randomly selected to be included in the study.

### Exposure, mediator and outcome measures

We used the Scottish Index for Multiple Deprivation (SIMD) 2006 to proxy individual-level socioeconomic position in this study.^24^ We used SIMD version 2006 as it was closest in date to cohort members’ years of birth. SIMD is a relative measure based on data zone-level (small areas of 500–1000 residents) deprivation across seven domains: income, employment, health, education, access to services, crime and housing.^24^ SIMD deciles, based on residential address at birth, were retrieved from birth registration files and supplemented from Scottish Birth Record/SMR02 where missing. SIMD deciles were split into groups for analyses indicating high (top 30% rank of SIMD scores), medium (middle 40% rank) and low (bottom 30%) socioeconomic deprivation.

The mediator was defined as having ≥1 hospital admission with a primary or secondary diagnosis of bronchiolitis during the first year of life, identified in SMR01 records by the International Classification of Diseases 10th Revision (ICD-10) code J21 for acute bronchiolitis. We included all J21 acute bronchiolitis subgroups in the mediator (J21.0 due to RSV, J21.1 due to human metapneumovirus, J21.8 due to other specified organisms, J21.9 unspecified) because specific bronchiolitis diagnoses are poorly coded in Scottish hospital admission data.^25^ It is estimated that almost 80% of admissions with a primary diagnoses of bronchiolitis among infants can be attributed to RSV.^26^

The outcome consisted of trajectory groups of asthma/wheeze (see Supplementary Material Part 1, available as Supplementary data at *IJE* online for an explanation of this term), determined through the latent growth curve modelling described below. To model trajectories, we first defined instances of asthma/wheeze at specific time points. For each year of age between 2 and 9 inclusive, children were defined as having asthma and/or wheeze if they had: ≥1 hospital admission in SMR01 with a main diagnosis, as defined by ICD-10 codes J45 for asthma, J46X for status asthmaticus or R06.2 for wheezing, and/or ≥4 dispensed prescriptions for any specified asthma medication (see Supplementary Table S1, available as Supplementary data at *IJE* online).

### Covariates

**Figure 1:**
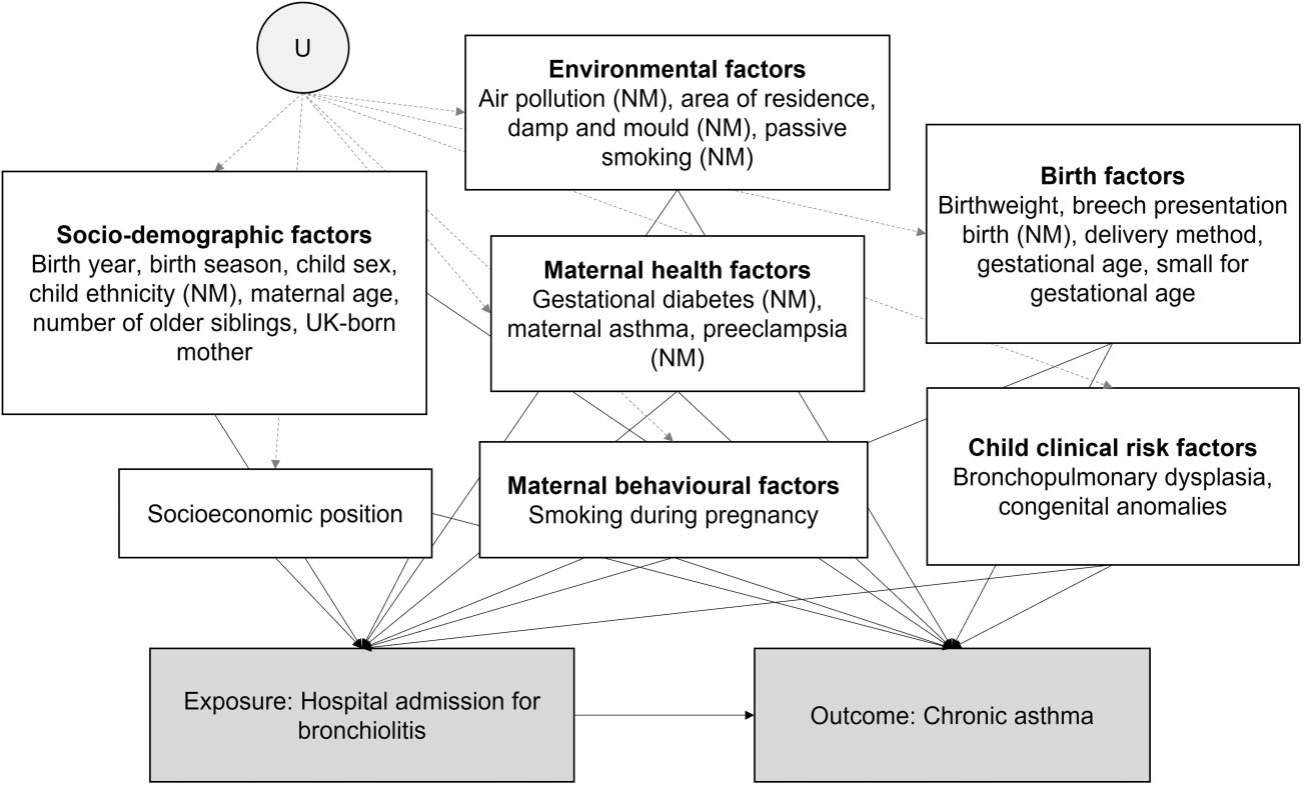
Assumed causal directed acyclic graph for the bronchiolitis admission (exposure in this graph, mediator in the final model) and chronic asthma (outcome) relationship. Confounders, grouped by type of risk factor, are listed in white boxes with NM indicating variables not measured in this study. U represents common unmeasured variables

Identification of the disparity measures described below invokes the assumption of no unmeasured confounding of the mediator-outcome relationship. We used an evidence synthesis approach, as proposed by Ferguson *et al*.,^27^ to construct our directed acyclic graph (DAG). See Supplementary Material Part 2 (available as Supplementary data at *IJE* online) for implementation of this approach. A simplified version of the derived DAG is shown in Figure 1 where we list the identified confounding variables, highlighting those not available in our study. Table 1 and Supplementary Material Part 3 (available as Supplementary data at *IJE* online) outline the included confounding variables.

**Table 1:** Descriptions of included confounders of the mediator-outcome relationship

Variable	Values	Source	Derived from
**Birth factors**			
Birthweight	Not applicable (variable used to derive SGA)	SMR02, SBR if missing	Birthweight (grams)
Delivery method	Vaginal, elective caesarean and emergency caesarean	SMR02	
Preterm birth	Born <37 weeks gestation (yes, no)	SMR02	Gestational age (weeks)
SGA	Birthweight falling below the 10th centile of the sex-, and gestational age-specific distributions^a^ (yes, no)	As above	Gestational age and birthweight
**Child health factors**			
Chronic condition	Presence of long-term condition, diagnosed before 6 months of age (yes, no), including bronchopulmonary dysplasia and congenital anomalies. See Supplementary Table S3 (available as Supplementary data at IJE online) for the full list of diagnoses	SBR, death record, SMR01 (child’s)	Clinical diagnoses
**Environmental factors**			
Area of residence^b^	Northern Scotland, central Scotland, Glasgow, Edinburgh, southern Scotland (see Supplementary Figure S2, available as Supplementary data at IJE online)	NRS birth registration	Postcode area (i.e. first two letters) of residential address
**Maternal behavioural factors**			
Mother’s smoking status	Self-reported smoking traditional cigarettes at around 8 to 12 weeks’ gestation (yes, no)	SMR02	
**Maternal health factors**			
Severe maternal asthma	≥1 hospital admission before their child’s birth that included a diagnosis of asthma including all subcategories (ICD-10 code J45) (yes, no)	SMR01 (mother’s)	Clinical diagnoses
**Sociodemographic factors**			
Birth season	Spring, summer, autumn, winter	NRS birth registration	Date of birth
Birth year^b^	2007, 2008	NRS birth registration	Date of birth
Child sex	Female, male	NRS birth registration	
Maternal age at birth	<20, 20–29, 30–39 and ≥40 years	SMR02	
Number of older siblings	0, 1, ≥2 siblings	SMR02	Parity
UK-born mother^b^	Yes, no	NRS birth registration	Mother’s country of birth

ICD-10 ,  International Classification of Diseases 10th revision; NRS ,  National Records of Scotland; SBR ,  Scottish Birth Record; SGA ,  small for gestational age; SIMD ,  Scottish Index for Multiple Deprivation; SMR01 ,  Scottish Morbidity Record 01; SMR02 ,  Scottish Morbidity Record 02.

a Distributions defined using LMS tables (Cole, Williams & Wright, 2001).

b Also included as confounders in the exposure-outcome relationship.

We also included additional variables related to missingness in confounders to be used in the imputation models outlined below: National Statistics Socio-Economic Classification (an occupation-based measure of social class), child’s postnatal intensive care stay, preterm-related complications and birth hospitals with high levels of missing values indicator (full description in Supplementary Material Part 3).

### Statistical analysis

First, we described cohort characteristics and missing data. The association between variables and the probability of missingness in at least one study variable was explored using multivariable logistic regression. There was a relatively high level of missing data across the cohort (10.3% of children had ≥1 missing value) and, prominently, we found that missingness was associated with observed data. Therefore, we imputed the missing values assuming missingness was at random, conditional on the covariates listed above (as well as the confounders, exposure, mediator and outcome).^28^

We used latent class growth analysis (LCGA), a statistical approach to detecting classes of individuals with a similar developmental trajectory, to model asthma/wheeze groups.^29^ Trajectory groups are not necessarily true phenotypic groups, but represent descriptions of the variation in individual trajectories, where the prevalence of each group is based on estimated posterior probabilities.^30^ The dichotomous outcomes of asthma/wheeze presence between ages 2 and 9 years were modelled using a mixture of logistic distributions, with age as the only explanatory variable. Based on previous research,^14^ we fitted LCGA models with three groups initially and added more groups in a stepwise manner to select the best fitting model (using criteria described in Supplementary Material Part 4, available as Supplementary data at *IJE* online). Due to the low frequencies of some of the identified asthma/wheeze groups, risk estimates and comparative disparity measures were carried out after recoding these groups into ‘chronic asthma’ versus ‘no/non-chronic asthma’ (further details in Results section). This classification is justified by the clinical difference between the non-chronic and chronic trajectories, whereby the former are most likely linked to early wheeze than asthma.^10^

Disparities in chronic asthma by SIMD group (per 1000 children) were defined as marginal risk differences and estimated by inverse probability weighting of a binomial regression model with weights expressed as function of year of birth, maternal country of birth and area of residence. To capture the proportion of socioeconomic disparity in asthma that would remain if hospitalization for bronchiolitis were prevented, we used counterfactual disparity measures (CDM).^31^ By setting the mediator to a predefined value (*m *=* *0 denoting absence of hospitalization for bronchiolitis), CDM captures the disparity in the outcome risk due to the exposure that would remain if the mediator were intervened upon and set to *m*, without intervening on the exposure. The formal definition and key assumptions to this approach are outlined in Supplementary Material Part 5 (available as Supplementary data at *IJE* online).

We used inverse probability weighting of marginal structural models to estimate CDM(*m *=* *0),^32^ with weights expressed as a function of the confounders detailed in Table 1. Results are displayed as the remaining risk differences in chronic asthma between high and medium SIMD versus low SIMD (per 1000 children) if no child had a hospital admission for bronchiolitis during infancy. Using the estimated marginal risk differences in chronic asthma, we also derived the proportion of disparity reduction attributable to elimination of bronchiolitis admissions as: Disparity reduction (%) was estimated as = (risk difference–CDM(*m* = 0))/risk difference × 100. To calculate confidence intervals for the estimates of CDM(*m *=* *0), risk difference and proportion of disparity eliminated, while addressing the presence of missing data in our dataset, we used single stochastic imputation using chained equations with 10 burn-in iterations as outlined by Micali *et al*.^33^^,^^34^ Imputation was directly followed by the CDM analysis, and both processes were repeated on 1000 bootstrap samples to estimate standard errors. To meet the missing at random assumption, we conditioned on all covariates from the substantive model, alongside the additional variables listed above (further details in Supplementary Material Part 5).

We used Mplus version 8.2 to implement LCGA, Stata 15 for descriptive data analysis and mediation analysis.^35^^,^^36^ The code for LCGA and mediation analyses can be found at [https://github.com/UCL-CHIG/SEP-bronchiolitis-asthma-study]. DAGitty was used to determine minimally sufficient adjustment sets for the identification of CDMs.^37^

## Results

### Cohort characteristics

**Figure 2:**
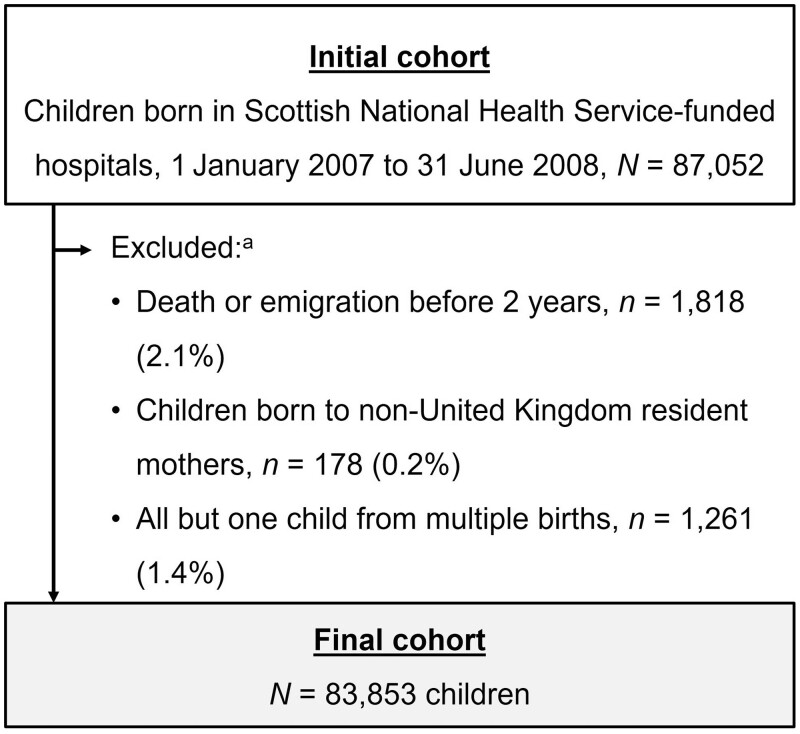
Flow diagram showing derivation of final study cohort; ^a^groups not mutually exclusive

Of the 83 853 children in the cohort after exclusions were applied (Figure 2, Table 2), 48.9% were female and 40.3% were resident in Edinburgh or Glasgow at birth. Of these, 5140 infants were born before 37 weeks’ gestation and 7442 were classified as small for gestational age (6.5% and 9.4% of those with available data, respectively). A total of 20 095 infants (25.3%) with a delivery method recorded were born via a caesarean section, and 16 600 mothers reported smoking traditional cigarettes during their pregnancy (21.9% of those with available data). Further, 3.2% of children (2675) were admitted to hospital at least once with a diagnosis of bronchiolitis in the first year of life and 9.3% (7819) met the study definition for asthma/wheeze at least once during follow-up. The mother’s delivery record linked for 79 500 (94.8%) of children, and the mean follow-up time from birth per child was 8.84 years (standard deviation 0.92). Of the cohort children, 2325 (2.8%) either emigrated or died by their ninth birthday (Supplementary Table S4, available as Supplementary data at *IJE* online).

**Table 2:** Characteristics of final study cohort (pre-imputation), number of children with missing data and estimated adjusted odds ratios for missingness, *N *=* *83 853

		Whole cohort		Missing data on ≥1 variable		
		*N*	%	Yes	%	aOR^b^ (95% CI)
Total		83 853	100.0	8632	100.0	
Exposure: SIMD	Low	21 991	26.2	2115	24.5	Reference
	Medium	31 738	37.8	2974	34.5	1.03 (0.97–1.10)
	High	30 124	35.9	3543	41.0	0.97 (0.90–1.04)
Outcome: Any asthma/wheeze^a^	No	76 034	90.7	7819	90.6	Reference
	Yes	7819	9.3	813	9.4	0.99 (0.92–1.08)
Mediator: ≥1 bronchiolitis admission	No	81 178	96.8	8404	97.4	Reference
	Yes	2675	3.2	228	2.6	0.92 (0.80–1.07)
Confounders:						
Birth season	Spring	27 967	33.4	3045	35.3	Reference
	Summer	19 180	22.9	1787	20.7	0.80 (0.75–0.86)
	Autumn	14 083	16.8	1479	17.1	0.90 (0.83–0.96)
	Winter	22 623	27.0	2321	26.9	0.91 (0.85–0.96)
Sex	Female	40 972	48.9	4212	48.8	Reference
	Male	42 881	51.1	4420	51.2	0.99 (0.95–1.04)
Chronic condition	No	80 498	96.0	8236	95.4	Reference
	Yes	3355	4.0	396	4.6	1.26 (1.13–1.41)
Area	Northern	10 903	13.0	566	6.6	Reference
	Central	23 785	28.4	1865	21.6	1.52 (1.37–1.67)
	Glasgow	19 516	23.3	3966	45.9	1.42 (1.28–1.58)
	Edinburgh	14 254	17.0	1144	13.3	1.55 (1.39–1.72)
	Southern	15 395	18.4	1091	12.6	1.22 (1.10–1.36)
Maternal county of birth	Non-UK	9061	10.8	1053	12.2	Reference
	UK	74 792	89.2	7579	87.8	0.88 (0.82–0.95)
Maternal age	<20	6019	7.2	560	6.5	
	20–29	36 099	43.1	1970	22.8	
	35–39	33 071	39.4	1523	17.6	
	>40	4311	5.1	226	2.6	
	Missing	4353	5.2	4353	50.4	
Severe maternal asthma	No	77 518	92.4	4177	48.4	
	Yes	1982	2.4	102	1.2	
	Missing	4353	5.2	4353	50.4	
Maternal smoking	No	59 093	70.5	371	4.3	
	Yes	16 600	19.8	101	1.2	
	Missing	8160	9.7	8160	94.5	
Siblings	0	37 158	44.3	2869	33.2	
	1	26 701	31.8	863	10.0	
	2+	15 337	18.3	243	2.8	
	Missing	4657	5.6	4657	54.0	
Delivery method	Vaginal	59 403	70.8	2951	34.2	
	Elective caesarean	8202	9.7	425	4.9	
	Emergency caesarean	11 893	14.2	901	10.4	
	Missing	4355	5.2	4355	50.5	
Preterm birth	No	74 302	88.6	3820	44.3	
	Yes	5140	6.1	401	4.6	
	Missing	4411	5.3	4411	51.1	
SGA	No	71 596	85.4	3330	38.6	
	Yes	7442	8.9	487	5.6	
	Missing	4815	5.7	4815	55.8	

CI, confidence intervals; SGA, small for gestational age, SIMD, Scottish Index for Multiple Deprivation.

a Children meeting any asthma/wheeze definition at ages between 2 and 9 years.

b Multivariable logistic regression comparing the likelihood of having missing data on ≥1 variable with no missing data (aOR = adjusted odds ratio). Also included in the missingness model and not shown in table: birth at hospital with high levels of missing values, aOR 8.99 (95% CI 8.33–9.70); later birth year, aOR 0.87 (95% CI 0.82–0.92); intensive care stay, aOR 1.18 (95% CI 1.08–1.29); prematurity-related complication, aOR 1.01 (95% CI 0.87–1.18); and National Statistics Socio-Economic Classification analytical classes.

Of cohort members, 8632 (10.3%) had missing data for at least one variable. Missingness was driven by infants without linked maternal records (4353, 5.2%, of the cohort) and missing data on smoking status, which was missing for an additional 3807 of the infants who were linked to mother’s records (9.7% missing smoking status in total). As shown in Table 2, missingness in any variable was more common in children with: postal areas in South Scotland, particularly Glasgow; earlier birth years; births between March and May; mothers born outside the UK; and intensive care stay after birth.

### Growth modelling

**Figure 3:**
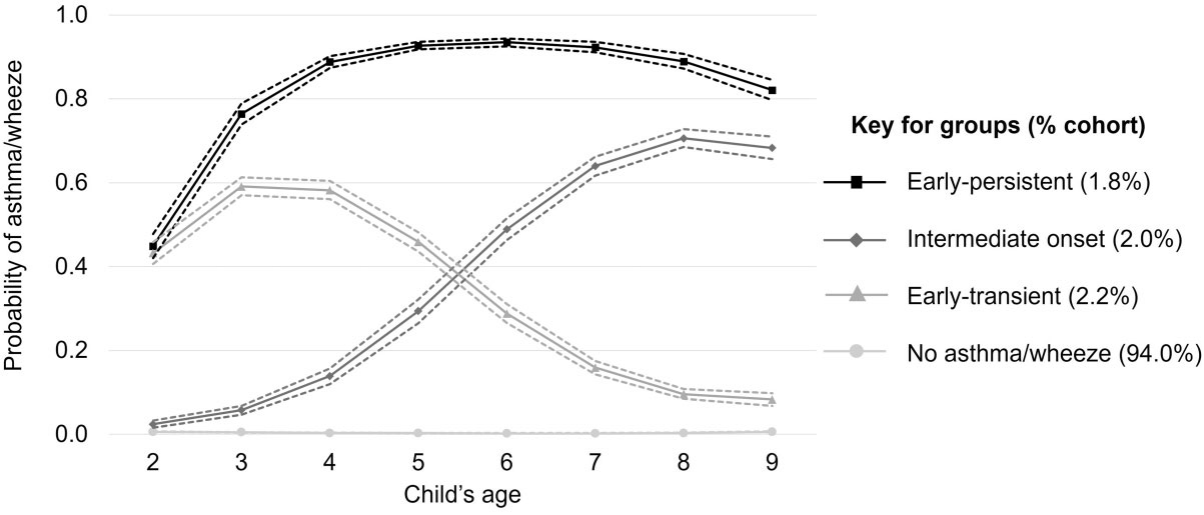
Estimated probabilities (and 95% confidence intervals) of experiencing asthma/wheeze at each time point for latent trajectory groups, derived from the posterior probability distributions of asthma/wheeze prevalence, estimated for the four-class latent class growth analysis model with cubic growth for age. The ‘no asthma/wheeze’ group has a consistent near 0 probability of reporting asthma/wheeze

The LCGA model that identified four groups and a cubic polynomial function for the association with age were selected as the most suitable overall according to fit indices (see Supplementary Table S5, available as Supplementary data at *IJE* online). Supplementary Figure 2 (available as Supplementary data at *IJE* online) displays average latent class probabilities by most likely class membership. The posterior distributions of asthma/wheeze prevalence at each time point by class are displayed in Figure 3. The groups can be summarized as: no asthma/wheeze, 94.0% of the cohort had none or (for a minority of the group) one instance of asthma/wheeze from ages 2 to 9 years; early-transient, 2.2% of the cohort had asthma/wheeze that had begun by age 2, had a peak prevalence at age 3 and dissipated by age 7; intermediate-onset, 2.0% of the cohort had asthma/wheeze that had begun by age 4 and had a peak prevalence at age 8, a year before follow-up ended; and early-persistent, 1.8% of the cohort had asthma/wheeze that had begun by age 2, had a peak prevalence at age 6, with ongoing symptoms at the end of follow-up. The distributions of all relevant variables by asthma/wheeze trajectories (Table 3) highlights differences in the proportion of children with ≥1 hospital admission for bronchiolitis by trajectory group: 10.8% (*n *=* *200) in the early-transient group, 9.0% (*n *=* *139) in the early-persistent group, 5.5% (*n *=* *90) in the intermediate-onset group and 2.8% (*n *=* *2206) in the no asthma/wheeze group.

**Table 3:** Exposure, outcome and confounders distribution by predicted asthma/wheeze trajectories groups (assigned according to the most likely class membership), *N *=* *83 853

		No asthma/wheeze		Early-transient		Early-persistent		Intermediate-onset	
		*n*	%	*n*	%	*n*	%	*n*	%
Total		78 803	94.0	1858	2.2	1547	1.8	1645	2.0
Exposure: SIMD	Low	20 962	95.3	380	1.7	306	1.4	352	1.6
	Medium	29 866	94.1	682	2.1	572	1.8	623	2.0
	High	27 975	92.9	791	2.6	668	2.2	670	2.2
Mediator: ≥1 bronchiolitis admission	No	76 597	94.3	1653	2.0	1408	1.7	1555	1.9
	Yes	2206	83.7	200	7.6	139	5.3	90	3.4
Confounders:									
Birth season	Spring	26 320	94.0	565	2.0	528	1.9	581	2.1
	Summer	17 967	93.8	441	2.3	367	1.9	373	1.9
	Autumn	13 239	94.2	317	2.3	238	1.7	262	1.9
	Winter	21 277	93.9	532	2.3	415	1.8	429	1.9
Sex	Female	39 007	95.2	726	1.8	579	1.4	661	1.6
	Male	39 796	92.8	1127	2.6	968	2.3	984	2.3
Chronic condition	No	75 808	94.1	1733	2.2	1437	1.8	1553	1.9
	Yes	3073	90.5	120	3.5	110	3.2	92	2.7
Area	Northern	10 244	94.2	222	2.0	196	1.8	207	1.9
	Central	22 459	94.3	526	2.2	402	1.7	419	1.8
	Glasgow	18 203	93.4	471	2.4	385	2.0	429	2.2
	Edinburgh	13 475	94.8	252	1.8	218	1.5	271	1.9
	Southern	14 342	93.2	382	2.5	347	2.3	316	2.1
Maternal birth country	Non-UK	8590	95.0	169	1.9	135	1.5	146	1.6
	UK	70 213	93.9	1684	2.3	1412	1.9	1499	2.0
Maternal age	<20	5595	93.0	148	2.5	118	2.0	158	2.6
	20–29	33 728	93.4	915	2.5	729	2.0	748	2.1
	30–39	31 285	94.7	634	1.9	555	1.7	579	1.8
	40+	4098	94.8	76	1.8	70	1.6	81	1.9
	Missing	4098	94.6	80	1.8	77	1.8	79	1.8
Severe maternal asthma	No	72 972	94.2	1692	2.2	1364	1.8	1472	1.9
	Yes	1734	86.0	82	4.1	107	5.3	94	4.7
	Missing	4098	94.6	80	1.8	77	1.8	79	1.8
Maternal smoking	No	55 714	94.3	1249	2.1	999	1.7	1105	1.9
	Yes	15 445	92.9	428	2.6	384	2.3	372	2.2
	Missing	7644	93.8	176	2.2	162	2.0	168	2.1
Siblings	0 siblings	34 831	93.7	873	2.3	688	1.9	767	2.1
	1 sibling	25 059	94.0	595	2.2	493	1.8	517	1.9
	2+ siblings	14 500	94.5	295	1.9	285	1.9	271	1.8
	Missing	4413	94.5	89	1.9	80	1.7	89	1.9
Delivery method	Vaginal	63 042	94.8	1293	1.9	1046	1.6	1148	1.7
	Elective	7723	93.9	178	2.2	158	1.9	168	2.0
	Emergency	11 111	93.1	302	2.5	268	2.2	252	2.1
	Missing	4098	94.6	80	1.8	77	1.8	79	1.8
Preterm	No	69 977	94.2	1579	2.1	1290	1.7	1441	1.9
	Yes	4649	90.3	195	3.8	179	3.5	123	2.4
	Missing	4177	94.6	80	1.8	77	1.7	81	1.8
SGA	No	67 298	94.0	1584	2.2	1287	1.8	1393	1.9
	Yes	6935	93.1	180	2.4	173	2.3	161	2.2
	Missing	4571	94.5	89	1.8	87	1.8	90	1.9

SGA, small for gestational age; SIMD, Scottish Index for Multiple Deprivation.

### Disparity analysis

We reclassified cohort members assigned to the early-persistent or intermediate-onset asthma/wheeze groups as ‘chronic asthma’ (3.8% of the cohort), and those assigned to no or early-transient asthma/wheeze as ‘non-chronic asthma’ (96.2%). The chronic asthma group experienced greater frequencies of high socioeconomic deprivation (41.9% vs 35.7%) and hospital admissions for bronchiolitis (7.2% vs 3.0%).

As shown in Table 4, the associational marginal risk of chronic asthma by age 9 is estimated as 30.8 per 1000 children (95% CI 28.3–33.3) in the low (least deprived) SIMD group at birth. This compares with 37.7 per 1000 in the medium SIMD group (95% CI 36.2–39.3) and 43.7 per 1000 in the high (most deprived) SIMD group (95% CI 41.3–46.1); the corresponding marginal risk differences are 7.0 (95% CI 3.7–10.3) and 13.0 (95% CI 9.6–16.4) additional cases of chronic asthma per 1000 children, respectively The CDM(*m *=* *0), suggests that if no children had a hospital admission for bronchiolitis in infancy, the counterfactual disparity between children in the medium and high compared with low SIMD group would be reduced to 5.5 per 1000 (95% 2.3–8.8) and 10.7 per 1000 (95% CI 7.3–14.0); in percentage terms these are reductions of 21.2% (95% CI 4.9–37.5) and 17.9% (95% CI 10.4–25.2), respectively.

**Table 4:** Estimated association risks and risk differences in chronic asthma risk and estimated counterfactual disparity measures by exposure level (*N *=* *83 853)

Exposure: SIMD group	Marginal risk per 1000 children (95% CI)^a^	Risk difference per 1000 children (95% CI)^b^	CDM(*m *=* *0) per 1000 children (95% CI)^b^	% disparity reduction (95% CI)^b^^,^^c^
Low	30.8 (28.3–33.3)	Reference	Reference	Reference
Medium	37.7 (36.2–39.3)	7.0 (3.7–10.3)	5.5 (2.3–8.8)	21.2 (4.9–37.5)
High	43.7 (41.3–46.1)	13.0 (9.6–16.4)	10.7 (7.3–14.0)	17.9 (10.4–25.4)

CDM, counterfactual disparity measure; CI, confidence intervals; SIMD, Scottish Index for Multiple Deprivation.

a Risk and absolute risk difference after accounting for differences in year of birth, maternal country of birth and area of residence between groups using inverse probability=weighting.

b 95% CIs calculated using the bootstrap with 1000 replications.

c Disparity reduction (%) was estimated as = (risk difference–CDM(*m* = 0))/risk difference × 100.

## Discussion

Using administrative data, this study was able to follow up more than 80 000 children until their 10th birthday to identify asthma/wheeze trajectories with minimal selection bias. We identified four different trajectories: early-transient (2.2% of the cohort), early-persistent (2.0%), intermediate-onset (1.8%) and no asthma/wheeze (94.0%). There was a socioeconomic gradient to asthma/wheeze group membership, with 43.7 per 1000 children born into areas of high socioeconomic deprivation assigned a chronic trajectory (early-persistent or intermediate-onset combined) compared with 30.8 per 1000 children born into areas with low socioeconomic deprivation. Using counterfactual disparity measures, we estimated that if bronchiolitis hospital admissions in infancy were eliminated, about one-fifth of the difference in the risk of chronic asthma between children from different socioeconomic groups in Scotland would be removed.

We used a range of clinical definitions to describe asthma/wheeze in our study with input from a clinician, in contrast to self- or parent-reported measures used elsewhere.^14^ This approach, paired with a cautious methodology to define symptoms, means there are likely to be fewer false-positives captured in this study.^38^ On the other hand, some children (with milder symptoms) will likely have been misclassified as not having had either bronchiolitis or asthma/wheeze in this study. Presentation and admission to hospital can be influenced by factors other than the severity of illness, including the availability of primary care, affordability of transport and child care, clinical decision making and (for paediatric patients) parental expectations.^39–41^ The social patterning of these influences may have introduced bias into the relationship between socioeconomic deprivation and bronchiolitis, although the direction of the bias needs to be determined. The addition of primary care and emergency department records in future administrative data studies may improve identification of all relevant cases of bronchiolitis and asthma/wheeze. Other studies have included measures of atopy in modelling of asthma trajectories.^12^^,^^42^ However, overlap between medication used to treat asthma and atopic conditions meant that the current dataset would not have allowed for this nuanced differentiation. In addition, although we used predetermined criteria to determine trajectories, these methods still have a degree of subjectivity.^12^ Although the class sizes were proportionally smaller than those found in other studies, there are similarities in the shape and relative size of asthma/wheeze trajectory groups modelled using other datasets.^13^^,^^15^

We had to take several pragmatic steps in this study, based on constraints of the methods and datasets used. We used an area-level measure of socioeconomic deprivation as a proxy for an individual-level indicator, potentially leading to an underestimation of the true individual-level socioeconomic effects.^43^ We encountered missing data, which was likely due to variation in recording practices by hospital, over time and by clinical need, but we dealt with this bias using imputation. We restricted the sample to children who were alive at the start of asthma/wheeze measurement, meaning that the findings are contingent on survival until the age of 2. This may have had the effect of biasing the CDM, most likely by underestimating it since the risk of mortality is higher among children with underlying illnesses and from the most socioeconomically deprived backgrounds.^44^ In addition, we were unable to separately examine the four asthma/wheeze trajectory groups in the CDM analysis because we encountered problems of stability of the estimates due to the small numbers in some subgroups. In the future, cohort studies from linked Scottish administrative data with births spanning several years will enable longer follow-up periods and a greater sample size for more nuanced analyses.

Two systematic reviews of the evidence published in 2020 commented on the high risk of confounding bias in observational studies looking at the relationship between RSV and subsequent chronic wheezing.^5^^,^^45^ We have used causal inference methods for a very targeted estimand, the CDM, with explicit discussions of the assumptions invoked to obtain estimates using our observational study. The estimation of CDM calls for a hypothetical intervention that reduces hospitalization due to bronchiolitis for all children to levels among those with low socioeconomic deprivation. This could be achieved for example by greater prevention efforts directed at children in poorer areas, for example ensuring high uptake of future RSV vaccines or monoclonal antibodies. There were several confounders of the mediator-outcome association identified in previous research which could not be included in this study. However, this uncontrolled confounding effect may be partially captured by the other variables that were included in the study. For example, gestational diabetes, pre-eclampsia and breech presentation birth are thought to, at least partially, influence offspring respiratory outcomes through delivery method and preterm birth.^46^^,^^47^ In addition, the extent of the residual confounding induced by these factors may have been moderated by controlling for socioeconomic position.

## Conclusion

This is the first time the pathways between socioeconomic position, bronchiolitis admissions and asthma have been explored using counterfactual methods. We estimate that about 20% of the disparity between socioeconomic groups may be attributable to an admission to hospital with bronchiolitis during infancy. Moreover, our work also highlights that at least 80% of the association between socioeconomic position and chronic asthma cannot be explained by hospital admission for bronchiolitis in infancy. This underscores the need to further investigate the causes of inequities in bronchiolitis admissions and wheeze/asthma. In the future, studies using administrative data could be enhanced by linkage to other datasets, for example the Department for Environments Food and Rural Affairs’ modelled background pollution data or prospectively designed cohort studies,^48^^,^^49^ which will enable more risk factors to be measured and further eliminate bias from calculations. This paper offers a guide to implement causal inference methods to carry out further counterfactual disparities analysis using these complex, linked health datasets.

## Ethics approval

The use of linked administrative health data from Scotland was approved by the Public Benefit and Privacy Panel, reference 1617–0224, and the South East Scotland Research Ethics Committee 02, reference 18/SS/0117.

## Supplementary Material

dyac193_Supplementary_Data

## Data Availability

This work uses data provided by patients and collected by the National Health Service (NHS) as part of their care and support. Authors do not have permission to share patient-level data. Data are available from Public Health Scotland [phs.edris@phs.scot] for researchers who meet the criteria for access to confidential data.
