# Supplementary material for: Socioeconomic position, bronchiolitis and asthma in children: counterfactual disparity measures from a national birth cohort study
Source: Int J Epidemiol. 2022 Sep 30;52(2):476–88. doi: 10.1093/ije/dyac193 (PMC10114124; doi:10.1093/ije/dyac193)
Supplement: dyac193_Supplementary_Data [file dyac193_supplementary_data.docx]

Supplementary File

1. Contents

[PART 1: Defining asthma 2](#_Toc113961236)

[PART 2: Identifying confounders of the mediator-outcome relationship 4](#_Toc113961237)

[PART 3: Defining confounders and additional variables 9](#_Toc113961238)

[PART 4: Latent class growth analysis model selection 14](#_Toc113961239)

[PART 5: Counterfactual disparity measure (CDM) 15](#_Toc113961240)

[PART 6: Results 20](#_Toc113961241)

[References 23](#_Toc113961242)

# PART 1: Defining asthma

**Explaining the term “asthma/wheeze”**

Diagnosing asthma in children younger than 5 is not recommended, in part due to the difficulty of using standardised tests in this population.^1^ Moreover, recurrent symptoms of wheeze is common in young children and is not necessarily an indicator of later asthma.^2^ As such, including symptoms between ages 2 and 4 in our study was intended to capture early wheeze patterns rather than asthma per se. Conversely, a child may have asthma, but currently be asymptomatic.^3^ It is for these reasons that we use the broader term “asthma/wheeze” rather than singular terms of asthma or wheeze to describe trajectories in this study. Age 2 was selected as the start of symptom follow-up to reduce the risk of capturing instances of bronchiolitis (the study mediator) in the outcome.

**Table S1.** Drugs used in our study to indicate presence of wheeze/asthma in children, defined using the British National Formulary for Children^4^

| BNF Chapter section | Conditions treated in children | Inclusions (age restriction applied) | Exclusions (age restriction applied) |
| --- | --- | --- | --- |
| 3.1 Bronchodilators | |  |  |
| 3.1.1.1 Selective beta2 agonists | Short acting beta2 agonists: Reversible airway obstruction | Salbutamol, Terbutaline |  |
|  | Long acting beta2 agonists: Croup – single dose of corticosteroids | Formoterol (age 6+), Salmeterol |  |
| 3.1.1.2 Other adrenoceptor agonists | Acute allergic and anaphylactic reactions, angioedema, cardiopulmonary resuscitation, severe croup | Orciprenaline sulfate (withdrawn from the market in 2010) | Adrenaline (epinephrine), Ephedrine (hydrochloride) |
| 3.1.2 Antimuscarinic bronchodilators | Reversible airway obstruction, rhinitis | Ipratropium bromide | Aclidinium bromide, Glycopyrronium, Indacaterol, Tiotropium, Umeclidinium |
| 3.1.3 Theophylline | Reversible airway obstruction, bronchospasm associated with chronic bronchitis, neonatal apnoea | Theophylline, Aminophylline |  |
| 3.1.5 Peak flow meters, inhaler devices and nebulisers |  | Standard range peak flow meter (age 6+), Low range peak flow meter (age 6+), Inhaler devices, Spacer devices |  |
| 3.2 Corticosteroids | |  |  |
| 3.2 Corticosteroids | Croup, bronchopulmonary dysplasia | Beclometasone dipropionate (age 2+), Budesonide (age 6 +), Ciclesonide (age 12+), Fluticasone propionate (age 4+), Mometasone furoate (age 12+) |  |
| 3.3 Cromoglicate and related therapy and leukotriene receptor antagonists | | | |
| 3.3.1 Cromoglicate and related therapy | Food allergy, allergic conjunctivitis, allergic rhinitis | Sodium cromoglicate (age 5+), Nedocromil Sodium (age 5+) |  |
| 3.3.2 Leukotriene receptor antagonists | Allergic rhinitis (15-19 years) | Montelukast | Zafirlukast (12+ years only) |
| 3.4.2 Allergen Immunotherapy |  | Omalizumab (6+) |  |
| 6.3.2 Glucocorticoid therapy | |  |  |
| 6.3.2 Glucocorticoid therapy | Croup, inflammation, ulcerative colitis, Crohn's disease | Hydrocortisone, Dexamethasone, Prednisone, Prednisolone  (in conjunction with hospital admission) | Betamethasone, Deflazacort, Methylprednisolone |

# PART 2: Identifying confounders of the mediator-outcome relationship

We used a modified version of Ferguson and colleagues’ evidence synthesis for constructing directed acyclic graphs (DAGs) to identify confounders of the bronchiolitis-asthma (mediator-outcome) relationship.^5^ This method guides DAG development from background knowledge and direct primary data analysis, whilst examining plausible causal assumptions.

Firstly, we mapped risk factors for bronchiolitis identified in the literature that were also associated with asthma into an initial graph. A directed edge was drawn from bronchiolitis (the mediator in our analysis) to asthma (the outcome), then the initial graph was saturated by drawing edges between all variables. To reduce the high volume of assessments required, we excluded variables at this initial stage that were not applicable in a UK context. The second stage, translation, involved applying causal theory to each relationship in the initial graph, including consideration of temporality, face-validity and prior theory/research. We then applied a counterfactual thought experiment; hypothesising the potential outcome that would occur if all individuals were set to receive the same counterfactual exposure (for example, if all infants were to be hospitalised for bronchiolitis). At this stage, a directed edge could be retained, reversed or removed. Again, the theory/evidence was considered in relation to the UK rather than the country where the published study was conducted. When all papers were assessed, a final index of all directed edges was created with similar variables combined to avoid repetition, as well as for purposes of parsimony (see Table S2).

The index in Table S2 was used to create the final DAG representing the relationship between bronchiolitis hospitalisation and asthma, and their common causes. The variables selected from this DAG to belong to the minimal adjustment set for identifying the causal relationship between mediator and outcome were: air pollution, area of residence, birth season, birth weight, birth year, breastfed, breech presentation birth, bronchopulmonary dysplasia, congenital anomalies, delivery method, child ethnicity, gestational age, gestational diabetes, maternal age, maternal asthma, maternal country of birth, maternal smoking, parity, preeclampsia, child sex and small for gestational age.

Table S2. Directed edge index

| # | Edge originates -> terminates | # | Edge originates -> terminates |
| --- | --- | --- | --- |
| 1 | Air pollution -> Asthma | **123** | Maternal asthma -> Breastfed |
| 2 | Air pollution -> Birth weight | **124** | Maternal asthma -> Breech presentation |
| 3 | Air pollution -> Breastfed | **125** | Maternal asthma -> Bronchiolitis admission |
| 4 | Air pollution -> Bronchiolitis admission | **126** | Maternal asthma -> Bronch. dysplasia |
| 5 | Air pollution -> Bronch. dysplasia | **127** | Maternal asthma -> Congenital anomalies |
| 6 | Air pollution -> Congenital anomalies | **128** | Maternal asthma -> Delivery method |
| 7 | Air pollution -> Gestational age | **129** | Maternal asthma -> Gestational age |
| 8 | Air pollution -> Gestational diabetes | **130** | Maternal asthma -> Gestational diabetes |
| 9 | Air pollution -> Preeclampsia | **131** | Maternal asthma -> Maternal smoking |
| 10 | Air pollution -> SGA | **132** | Maternal asthma -> Preeclampsia |
| 11 | Area of residence -> Air pollution | **133** | Maternal asthma -> SGA |
| 12 | Area of residence -> Asthma | **134** | Maternal country of birth -> Area of residence |
| 13 | Area of residence -> Bronchiolitis admission | **135** | Maternal country of birth -> Asthma |
| 14 | Area of residence -> Bronch. dysplasia | **136** | Maternal country of birth -> Breastfed |
| 15 | Area of residence -> Damp and mould | **137** | Maternal country of birth -> Breech presentation |
| 16 | Area of residence -> Delivery method | **138** | Maternal country of birth -> Bronchiolitis admission |
| 17 | Area of residence -> Maternal asthma | **139** | Maternal country of birth -> Bronch. dysplasia |
| 18 | Area of residence -> Passive smoking | **140** | Maternal country of birth -> Child ethnicity |
| 19 | Area of residence -> SGA | **141** | Maternal country of birth -> Congenital anomalies |
| 20 | Birth season -> Air pollution | **142** | Maternal country of birth -> Damp and mould |
| 21 | Birth season -> Asthma | **143** | Maternal country of birth -> Delivery method |
| 22 | Birth season -> Birth weight | **144** | Maternal country of birth -> Gestational age |
| 23 | Birth season -> Bronchiolitis admission | **145** | Maternal country of birth -> Gestational diabetes |
| 24 | Birth season -> Gestational age | **146** | Maternal country of birth -> Maternal age |
| 25 | Birth season -> Maternal smoking | **147** | Maternal country of birth -> Maternal ethnicity |
| 26 | Birth season -> Preeclampsia | **148** | Maternal country of birth -> Maternal smoking |
| 27 | Birth season -> SGA | **149** | Maternal country of birth -> Parental SEP |
| 28 | Birth weight -> Asthma | **150** | Maternal country of birth -> Parity |
| 29 | Birth weight -> Breech presentation birth | **151** | Maternal country of birth -> Passive smoking |
| 30 | Birth weight -> Bronchiolitis admission | **152** | Maternal country of birth -> Preeclampsia |
| 31 | Birth weight -> Bronch. dysplasia | **153** | Maternal country of birth -> SGA |
| 32 | Birth weight -> Delivery method | **154** | Maternal ethnicity -> Area of residence |
| 33 | Birth weight -> SGA | **155** | Maternal ethnicity -> Birth weight |
| 34 | Birth year -> Air pollution | **156** | Maternal ethnicity -> Breastfed |
| 35 | Birth year -> Asthma | **157** | Maternal ethnicity -> Breech presentation |
| 36 | Birth year -> Birth weight | **158** | Maternal ethnicity -> Bronch. dysplasia |
| 37 | Birth year -> Breastfed | **159** | Maternal ethnicity -> Child ethnicity |
| 38 | Birth year -> Breech presentation birth | **160** | Maternal ethnicity -> Congenital anomalies |
| 39 | Birth year -> Bronchiolitis admission | **161** | Maternal ethnicity -> Damp and mould |
| 40 | Birth year -> Bronch. dysplasia | **162** | Maternal ethnicity -> Delivery method |
| 41 | Birth year -> Congenital anomalies | **163** | Maternal ethnicity -> Gestational age |
| 42 | Birth year -> Delivery method | **164** | Maternal ethnicity -> Gestational diabetes |
| 43 | Birth year -> Gestational age | **165** | Maternal ethnicity -> Maternal age |
| 44 | Birth year -> Gestational diabetes | **166** | Maternal ethnicity -> Maternal asthma |
| 45 | Birth year -> Maternal age | **167** | Maternal ethnicity -> Maternal smoking |
| 46 | Birth year -> Maternal asthma | **168** | Maternal ethnicity -> Parental SEP |
| 47 | Birth year -> Maternal smoking | **169** | Maternal ethnicity -> Parity |
| 48 | Birth year -> Parental SEP | **170** | Maternal ethnicity -> Passive smoking |
| 49 | Birth year -> Parity | **171** | Maternal ethnicity -> Preeclampsia |
| 50 | Birth year -> Preeclampsia | **172** | Maternal ethnicity -> SGA |
| 51 | Birth year -> SGA | **173** | Maternal smoking -> Air pollution |
| 52 | Breastfed -> Asthma | **174** | Maternal smoking -> Area of residence |
| 53 | Breastfed -> Bronchiolitis admission | **175** | Maternal smoking -> Asthma |
| 54 | Breech presentation birth -> Asthma | **176** | Maternal smoking -> Birth weight |
| 55 | Breech presentation -> Breastfed | **177** | Maternal smoking -> Breastfed |
| 56 | Breech presentation -> Bronchiolitis admission | **178** | Maternal smoking -> Breech presentation |
| 57 | Breech presentation -> Bronch. dysplasia | **179** | Maternal smoking -> Bronchiolitis admission |
| 58 | Breech presentation -> Delivery method | **180** | Maternal smoking -> Bronch. dysplasia |
| 59 | Breech presentation -> Preeclampsia | **181** | Maternal smoking -> Congenital anomalies |
| 60 | Bronchiolitis admission -> Asthma | **182** | Maternal smoking -> Delivery method |
| 61 | Bronch. dysplasia -> Asthma | **183** | Maternal smoking -> Gestational age |
| 62 | Bronch. dysplasia -> Breastfed | **184** | Maternal smoking -> Gestational diabetes |
| 63 | Bronch. dysplasia -> Bronchiolitis admission | **185** | Maternal smoking -> Passive smoking |
| 64 | Child ethnicity -> Asthma | **186** | Maternal smoking -> Preeclampsia |
| 65 | Child ethnicity -> Birth weight | **187** | Maternal smoking -> SGA |
| 66 | Child ethnicity -> Bronchiolitis admission | **188** | Parental SEP -> Asthma |
| 67 | Child ethnicity -> Bronch. dysplasia | **189** | Parental SEP -> Birth weight |
| 68 | Child ethnicity -> Congenital anomalies | **190** | Parental SEP -> Breastfed |
| 69 | Child ethnicity -> Delivery method | **191** | Parental SEP -> Breech presentation birth |
| 70 | Child ethnicity -> Gestational age | **192** | Parental SEP -> Bronchiolitis admission |
| 71 | Child ethnicity -> SGA | **193** | Parental SEP -> Bronch. dysplasia |
| 72 | Congenital anomalies -> Asthma | **194** | Parental SEP -> Congenital anomalies |
| 73 | Congenital anomalies -> Birth weight | **195** | Parental SEP -> Damp and mould |
| 74 | Congenital anomalies -> Breech presentation birth | **196** | Parental SEP -> Delivery method |
| 75 | Congenital anomalies -> Bronchiolitis admission | **197** | Parental SEP -> Gestational age |
| 76 | Congenital anomalies -> Bronch. dysplasia | **198** | Parental SEP -> Gestational diabetes |
| 77 | Congenital anomalies -> Delivery method | **199** | Parental SEP -> Maternal age |
| 78 | Congenital anomalies -> Gestational age | **200** | Parental SEP -> Maternal asthma |
| 79 | Congenital anomalies -> SGA | **201** | Parental SEP -> Maternal smoking |
| 80 | Damp and mould -> Asthma | **202** | Parental SEP -> Parity |
| 81 | Damp and mould -> Bronchiolitis admission | **203** | Parental SEP -> Passive smoking |
| 82 | Delivery method -> Asthma | **204** | Parental SEP -> Preeclampsia |
| 83 | Delivery method -> Breastfed | **205** | Parental SEP -> SGA |
| 84 | Delivery method -> Bronchiolitis admission | **206** | Parity -> Asthma |
| 85 | Delivery method -> Bronch. dysplasia | **207** | Parity -> Birth weight |
| 86 | Gestational age -> Asthma | **208** | Parity -> Breastfed |
| 87 | Gestational age -> Birth weight | **209** | Parity -> Breech presentation birth |
| 88 | Gestational age -> Breastfed | **210** | Parity -> Bronchiolitis admission |
| 89 | Gestational age -> Breech presentation birth | **211** | Parity -> Bronch. dysplasia |
| 90 | Gestational age -> Bronchiolitis admission | **212** | Parity -> Congenital anomalies |
| 91 | Gestational age -> Bronch. dysplasia | **213** | Parity -> Delivery method |
| 92 | Gestational age -> Delivery method | **214** | Parity -> Gestational age |
| 93 | Gestational diabetes -> Area of residence | **215** | Parity -> Gestational diabetes |
| 94 | Gestational diabetes -> Asthma | **216** | Parity -> Maternal smoking |
| 95 | Gestational diabetes -> Birth weight | **217** | Parity -> Preeclampsia |
| 96 | Gestational diabetes -> Breastfed | **218** | Parity -> SGA |
| 97 | Gestational diabetes -> Breech presentation birth | **219** | Passive smoking -> Asthma |
| 98 | Gestational diabetes -> Bronchiolitis admission | **220** | Passive smoking -> Bronchiolitis admission |
| 99 | Gestational diabetes -> Bronch. dysplasia | **221** | Preeclampsia -> Asthma |
| 100 | Gestational diabetes -> Congenital anomalies | **222** | Preeclampsia -> Birth weight |
| 101 | Gestational diabetes -> Delivery method | **223** | Preeclampsia -> Bronchiolitis admission |
| 102 | Gestational diabetes -> Gestational age | **224** | Preeclampsia -> Bronch. dysplasia |
| 103 | Gestational diabetes -> Preeclampsia | **225** | Preeclampsia -> Congenital anomalies |
| 104 | Gestational diabetes -> SGA | **226** | Preeclampsia -> Delivery method |
| 105 | Maternal age -> Asthma | **227** | Preeclampsia -> Gestational age |
| 106 | Maternal age -> Birth weight | **228** | Preeclampsia -> SGA |
| 107 | Maternal age -> Breastfed | **229** | SGA -> Asthma |
| 108 | Maternal age -> Breech presentation birth | **230** | SGA -> Breastfed |
| 109 | Maternal age -> Bronchiolitis admission | **231** | SGA -> Breech presentation birth |
| 110 | Maternal age -> Bronch. dysplasia | **232** | Sex -> Asthma |
| 111 | Maternal age -> Congenital anomalies | **233** | Sex -> Birth weight |
| 112 | Maternal age -> Delivery method | **234** | Sex -> Breastfed |
| 113 | Maternal age -> Gestational age | **235** | Sex -> Breech presentation birth |
| 114 | Maternal age -> Gestational diabetes | **236** | Sex -> Bronchiolitis admission |
| 115 | Maternal age -> Maternal asthma | **237** | Sex -> Bronch. dysplasia |
| 116 | Maternal age -> Maternal smoking | **238** | Sex -> Congenital anomalies |
| 117 | Maternal age -> Parity | **239** | Sex -> Delivery method |
| 118 | Maternal age -> Passive smoking | **240** | Sex -> Gestational age |
| 119 | Maternal age -> Preeclampsia | **241** | Sex -> Gestational diabetes |
| 120 | Maternal age -> SGA | **242** | Sex -> Preeclampsia |
| 121 | Maternal asthma -> Asthma | **243** | Sex -> SGA |
| 122 | Maternal asthma -> Birth weight |  |  |

Bronch. dysplasia= Bronchopulmonary dysplasia

# PART 3: Defining confounders and additional variables

**Confounders**

*Socio-demographic factors*

Child sex (female/male), date of birth, mother’s country of birth (dichotomised into UK or non-UK born) and postcode area were retrieved from the child’s birth registration file. Year and month of birth, used as an indicator of birth season, were derived from date of birth. The 16 postcode areas in Scotland were grouped to make 5 broader areas with similar population sizes as shown in Figure S2: northern Scotland (Harris, Inverness, Wick and Shetland), central Scotland (Aberdeen, Dundee, Falkirk, Kirkcaldy, Paisley and Perth), Glasgow, Edinburgh and southern Scotland (Douglas, Kilmarnock, Motherwell and Galashiels).

**Figure S1.** Map of Scotland, by postcode area: key showing 5 grouping used in this study


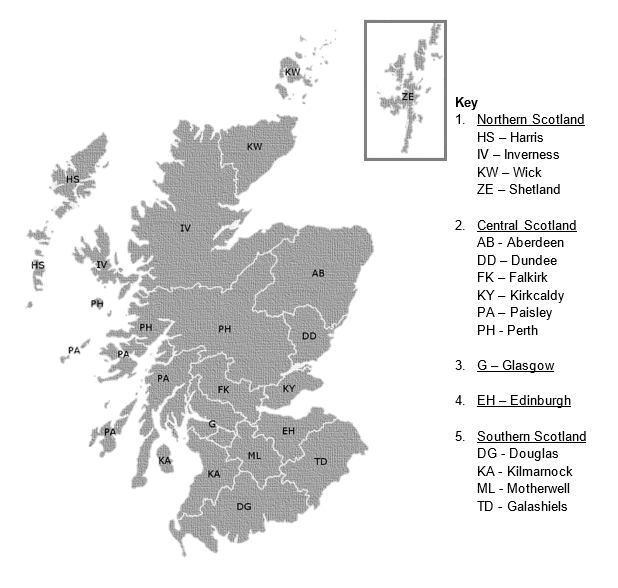


*Maternal factors*

The mother’s delivery record provided data on method of delivery (categorised into vaginal, elective caesarean and emergency caesarean), maternal age, parity, mother’s smoking status during pregnancy, gestational age in weeks and birthweight in grammes. Birthweight was supplemented with information from SBR where missing.

Maternal age was coded into a four-category variable (< 20, 20–29, 30–39 and ≥40 years) and parity (count of previous births) was used to create a number of older siblings variable that was split into groups (0, 1 and 2 or more siblings). We created an indicator of severe maternal asthma (yes/no), for mothers who had one or more hospital admission before their child’s birth that included a diagnosis of asthma (ICD-10 code J45, including all subcategories).

*Birth factors*

Gestational age was categorised as preterm (<37 weeks) or non-preterm (≥37 weeks). SGA (yes/no) was defined by birthweights falling below the 10^th^ centile of the sex-, and gestational age-specific distributions (using LMS tables).^6^

*Child clinical risk factors*

We created an indicator of chronic conditions, including congenital anomalies and bronchopulmonary dysplasia, using the list of International Classification of Diseases 10th revision ICD-10 codes defined by Kristensen and colleagues (see Table S2).^7^ Children were categorised as having a chronic condition (yes or no) if they had any of these diagnoses recorded in their birth record, death record or any hospital admission before 6 months of age.

*Maternal behavioural factors*

Mothers self-reported smoking status, recorded at around 8-12 weeks gestation, was treated as a binary variable with yes signifying mothers smoked traditional cigarettes (i.e. non-e-cigarettes) during their pregnancy.

**Covariates used in the imputation model**

Imputation models were used to deal with missingness affecting several of the confounders (shown in Table 1 in the main text). They included the following covariates (as well as the confounders, exposure, mediator and outcome):

- The 9-class National Statistics Socio-Economic Classification (an employment based measure of social class), based on father’s occupation for married/cohabiting couples and on mother’s occupation for all other birth registration types,^8^ retrieved from NRS birth registration files.
- A binary intensive care stay variable, with yes indicating any postnatal intensive or high dependency care recorded in the child’s SBR.
- Any child admitted to hospital diagnosed with any of the following ICD-10 codes in the first few months of life were recorded as having a prematurity-related complication: H35.1 (retinopathy of prematurity); P01.1 (newborn affected by premature rupture of membranes); P07.0 (extremely low BW <1000g) and P07.2 (extreme prematurity, <28 w); P27.0, P27.1, P27.8 and P27.9 (chronic respiratory diseases originating in the perinatal period); P59.0 (neonatal jaundice associated with preterm delivery); and P61.2 (anemia of prematurity). This indicator was used in addition to the chronic conditions indicator discussed above specifically for imputation purposes.
- A binary “birth hospitals with high missingness” indicator, with yes representing birth hospitals with missingness (i.e. proportion of children with at least one missing value across study covariates) ≥2 standard deviations above the overall birth hospital mean.
- We retrieved birth hospital from the child’s birth file and supplemented with the mother’s delivery file where missing.

**Table** **S3.** ICD-10 codes defining chronic conditions,^a^ from Kristensen et al.^7^

| ICD-10 code/s | | Condition/s |
| --- | --- | --- |
| Malformations of the respiratory system | | |
|  | Q30-Q34 | Congenital malformations of the respiratory system |
|  | Q35-Q37 | Cleft lip and cleft palate |
| Other conditions associated with respiratory symptoms | | |
|  | E84 | Cystic fibrosis |
|  | P27 | Bronchopulmonary dysplasia |
|  | Q39 | Congenital malformations of the esophagus |
|  | Q79.0 | Congenital diaphragmatic hernia |
| Neuromuscular disease | | |
|  | G60 | Hereditary and idiopathic neuropathy |
|  | G70.2 | Congenital and developmental myasthenia |
|  | G80 | Cerebral palsy |
|  | P94 | Disorders of muscle tone of newborn |
|  | Q01 | Encephalocele |
|  | Q02 | Microcephalus |
|  | Q03 | Congenital hydrocephalus |
|  | Q04 | Other congenital malformations of brain |
|  | Q05-Q07 | Spina bifida, malformations of the spinal cord and nervous system |
|  | G12 | Spinal muscular atrophy and related syndromes |
|  | G71.0–G71.3 | Muscular dystrophy |
| Congenital diseases of the heart and urinary system | | |
|  | D80–D82 | Congenital immunodeficiencies |
|  | E70.0–E73.0 | Inborn errors of metabolism |
|  | E74.0–E83.9 | Other metabolic disorders |
|  | I27.0 | Primary pulmonary hypertension |
|  | N07 | Hereditary nephropathy, not elsewhere classified |
|  | N13 | Malformations of the urinary system |
|  | N25 | Disorders resulting from impaired renal tubular function |
|  | P35 | Congenital viral diseases |
|  | Q20-Q26 | Congenital heart disease |
|  | Q40–Q44 | Congenital malformations of the digestive system |
|  | Q45.0–Q45.3 | Other congenital malformations of digestive system |
|  | Q60–Q64 | Congenital malformations of the urinary system |
| Chromosomal abnormalities not elsewhere classified | | |
|  | Q90 | Down syndrome |
|  | Q91-Q99 | Other chromosomal abnormalities |
| Others | | |
|  | Q79.2, Q79.3 | Exomphalos and gastroschisis |
|  | Q86.0 | Fetal alcohol syndrome |

^a^Children were categorised as having a chronic condition (yes or no) if they had any of these diagnoses recorded in their birth record, death record or any hospital admission before 6 months of age.

# PART 4: Latent class growth analysis model selection

Based on the number of classes identified in previous research, we applied 3 groups in a latent class growth analysis (LCGA) model initially and added more groups (up to a maximum of 6) in a step-wise manner.^9^ For each number of classes, we compared models with linear and non-linear parametrizations of the association with age. Model estimation was repeated with 500 random starting value sets to avoid local solutions, which occurs “where during curve estimation a largest value (maximum) or smallest value (minimum) that a function takes is identified for only a given area on that curve, but that is not necessarily the largest or smallest value for the entire curve (i.e., the global minimum or maximum)”.^10^ ^p.203^

We used the Bayesian information criterion values (BIC; a lower score indicating better model fit) and the bootstrapped likelihood ratio test (BLRT; obtained using the TECH14 option in Mplus) to guide model selection. In simulation studies, the BIC and BLRT have been shown to be the two best performing indices/tests to determine the number of classes in mixture modelling.^11,12^ The null hypothesis of the BLRT is that the model with $k-1$ classes fits the data as well as the model with $k$ classes, thus a low *p*-value points towards the $k$ class solution being a better fit of the model. We also required the final model to have latent classes that included at least 1% of the sample and an entropy value >0.8.^13,14^ The entropy value, which ranges from 0 to 1, indicates separation of classes.^15^ A lower entropy value implies poorer separation of classes which, in this case, indicates that there is uncertainty around membership of trajectory groups.

# PART 5: Counterfactual disparity measure (CDM)

**Formal definition**

Formally, our objective can be defined as: CDM(*m*=0)=E[*Y*(0)|*A*=a]-E[*Y*(0)|*A*=a*], where *A* = SIMD group (a if high/medium, a* if low), where *M* is a dummy indicator of ≥1 hospital admissions for bronchiolitis in the first year of life (1 if yes, 0 if no), *Y* is chronic asthma trajectory group (1 is yes, 0 if no), and Y(0) is the potential trajectory group had M been set to take the value 0. By setting the mediator to a predefined value (e.g. *m*=0), CDM captures the proportion of outcome disparity due to the exposure that would remain if a mediator (i.e. bronchiolitis admissions) were intervened upon and set to a chosen value m, without intervening on *A*.^16^ This captures the contrast between the potential risks of chronic asthma under high/medium compared to low levels of socioeconomic deprivation, were no infants to have a hospital admission for bronchiolitis in infancy.

**Assumptions for causal inference**

The assumptions usually required to identify and estimate causal effects from observational data are no interference, consistency, positivity and, when using propensity score-based methods as in this study, conditional exchangeability.^17^ In the context of CDMs, these assumptions are applicable to the mediator-outcome relationship, but not the exposure-mediator or exposure-outcome relationship. This because we wish only to characterise the overall disparity with the CDM, thus the statistical association between exposure and outcome carries relevant information that is disparity-related but not necessarily causal.^18^

In our study, the assumption of no interference means that one child’s potential outcome (of asthma) is not dependent on another child’s hospitalisation for bronchiolitis.^17,19^ Given that the causes of asthma are complex and manifold, but mostly dependent on one’s own levels of exposure, we assume that interference is not violated in this study. Consistency requires the intervention on the exposure (here, the study mediator, hospitalisation due to bronchiolitis) to have been defined with enough precision that any variation in that exposure does not lead to a different outcome.^20^ Bronchiolitis is defined in our study by a diagnosis of acute bronchiolitis (J21 including all subgroups) during hospital admission. This diagnosis is clinical, based on typical and common symptoms ascertained through patient history and clinical examination.^21^ More cases of bronchiolitis may have been captured if the definition was extended to other diagnoses, such as “viral infection, unspecified” (ICD-10 code B34. 9), and to young children older than 1 year; however, this would have lessened the specificity of the definition. Our stricter definition gives us more confidence that the consistency assumption is met in our study.

With regards to the positivity assumption, this would require that within each stratum defined by the mediator-outcome confounders there is a non-zero probability that a child is hospitalised for bronchiolitis. Based on the large number of children in this study and small number of strata (particularly after the outcome, chronic asthma, is dichotomised) it is justified to assume that the positivity assumption is upheld.^17^

Through our study DAG for the relationship between bronchiolitis admission and asthma (see Figure S1), we have outlined the many confounders required for the conditional exchangeability assumption (i.e. exchangeable conditional on a set of covariates *C*) to be met. Some of these covariates are unavailable in the study dataset. One excluded confounder, breastfeeding, has been shown to be a protective factor for bronchiolitis and asthma, meaning its exclusion may lead to an underestimation of the mediating effect of bronchiolitis.^9^ All others, prominently outdoor air pollution and presence of damp/mould in the dwelling, are positively associated with both bronchiolitis and asthma incidence, conversely potentially leading to an overestimation of the mediating effect.^22,23^ However, the uncontrolled confounding effect of some unmeasured confounders may be partially captured by the other variables that were included in the study. For example, gestational diabetes, pre-eclampsia and breech presentation birth are thought to, at least partially, influence offspring respiratory through delivery method and preterm birth.^24,25^ In addition, the extent of the residual confounding induced by these factors may have been moderated by controlling for socioeconomic position.

**Model specification**

Inverse probability weighting (IPW) of marginal structural models can be used to estimate CDMs on the assumption that the mediator model is correctly specified as a function of all mediator-outcome confounders (in addition to assumptions of no interference, consistency, positivity and conditional exchangeability, see above).^18^

As shown in Web Box 1, to estimate CDM(*m*=0) using IPW estimation of marginal structural models, we began by generating weights from the exposure model using multinomial logistic regression and from the mediator model using logistic regression. We then fitted a weighted regression model of the outcome against the exposure, the mediator and the interaction between the exposure and mediator. The estimated coefficient for the mediator in this model represents the estimated CDM(*m*=0). Area of residence, year and maternal country of birth were included in the propensity score models that generated the weights as confounders of the exposure-outcome relationship. Area of residence, birth season birth year, chronic condition, delivery method, gestational age, maternal age, severe maternal asthma, maternal country of birth, maternal smoking, number of older siblings, SGA and child sex were included as confounders of the mediator-outcome relationship. Birthweight, which was available in the dataset and identified as a potential confounder, was not included due to its high correlation with gestational age and SGA.

Although confounders of the exposure-outcome relationship are not required when estimating the CDMs, they can be included to produce comparisons adjusted for these variables.^18^ We therefore quantified the extent of the exposure-outcome association by estimating the marginal risk of chronic asthma by SIMD group using a logistic regression model of the outcome against the exposure using inverse probability weighting. The weights used for this are found by logistic regression modelling of the exposure that includes the exposure-outcome confounders, year of birth, maternal country of birth and area of residence.

Supplementary Box 1. Steps for estimating CDM(*m*=0) using IPW estimation of marginal structural models with a categorical exposure and binary mediator

Step 1a. Obtain predicted exposure probabilities from a multinomial model for *X*, unadjusted and adjusted for the exposure-outcome confounders, $p\left( X=x \right)$ and $p_{adj}\left( X=x \right)$, respectively.

Step 1b. Generate the weights corresponding to each level of the exposure

$$w\left( X=x \right)=\frac{p\left( X=x \right)}{p_{adj}\left( X=x \right)}$$

Step 2a. Obtain predicted mediator probabilities (unadjusted and adjusted for confounders),

$${p\left( M=1 \right)=\left\{ 1+\exp\left[ -\alpha_{0} \right] \right\}}^{-1}$$

$${p_{adj}\left( M=1 \right)=\left\{ 1+\exp\left[ -\alpha_{0}-\sum_{j=2}^{3} \alpha_{1j}I_{X=j}-\alpha{'_{2}\boldsymbol{C}}_{XY}-\alpha{'_{3}\boldsymbol{C}}_{MY} \right] \right\}}^{-1}$$

where $I_{X=j}$ is an indicator of whether the exposure *X* takes value j, ***C****_XY_* is a vector of exposure-outcome confounders, ***C****_MY_* a vector of mediator-outcome confounders, bold indicates matrices and dashes (‘) indicate transposition of matrices.

Step 2b. Generate the weights for the mediator using the predicted values from these models, where the weights depend on the observed level of the mediator, e.g.

$$w(M=0)=\frac{p\left( M=0 \right)}{p_{adj}\left( M=0 \right)}$$

Step 3. Generate overall weights by multiplying the exposure and mediator weights:

$$ipw=w\left( X=x \right)w\left( M=m \right)$$

Step 4. Fit a weighted regression model of the outcome against the exposure, the mediator and the interaction between the exposure and mediator, using the *ipw* weights:

$$E_{ipw}\left( Y \right)=\theta_{0}+\theta_{1}X+\theta_{2}M+\theta_{1}XM$$

**Imputation**

Partially observed covariates were imputed using linear regression (for birthweight, gestational age and maternal age), logistic regression (for severe maternal asthma and smoking status) and multinomial logistic regression (for number of older siblings and delivery method) models. Following each imputation, preterm birth, maternal age groups, sibling groups and SGA were defined using the relevant variables.

# PART 6: Results

**Table S4.** Observed completeness and frequency of asthma/wheeze symptoms, by age of child

| Age | Completeness of asthma/wheeze indicator | | | Asthma/  wheeze symptoms *N* | % of cohort  (*N* = 83,853) |
| --- | --- | --- | --- | --- | --- |
|  | **Complete child record *N*** | **Child died or emigrated *N*** | **% of cohort**  **(*N* = 83,853)** |  |  |
| 2 | 83,853 | 0 | 0.0% | 2,212 | 2.64 |
| 3 | 83,298 | 555 | 0.7% | 2,771 | 3.30 |
| 4 | 82,783 | 1,070 | 1.3% | 3,209 | 3.83 |
| 5 | 82,431 | 1,422 | 1.7% | 3,190 | 3.80 |
| 6 | 82,182 | 1,671 | 2.0% | 3,074 | 3.67 |
| 7 | 81,959 | 1,894 | 2.3% | 3,093 | 3.69 |
| 8 | 81,735 | 2,118 | 2.5% | 3,018 | 3.60 |
| 9 | 81,528 | 2,325 | 2.8% | 3,151 | 3.76 |

Table S5. Model fit indices for latent class growth analysis on asthma/wheeze symptoms (selected model emboldened)

|  |  | **LL** | **BIC** | **Entropy** | **VLMR**  **(*p*)** | **BRLT**  **(*p*)** | **Sample size per class^a^** | | | | | | | | | | | |
| --- | --- | --- | --- | --- | --- | --- | --- | --- | --- | --- | --- | --- | --- | --- | --- | --- | --- | --- |
|  |  |  |  |  |  |  | **Class 1** | | **Class 2** | | **Class 3** | | **Class 4** | | **Class 5** | | **Class 6** | |
|  |  |  |  |  |  |  | **N** | **%** | **N** | **%** | **N** | **%** | **N** | **%** | **N** | **%** | **N** | **%** |
| **LCGA** |  |  |  |  |  |  |  |  |  |  |  |  |  |  |  |  |  |  |
| **3-class** | **Linear** | -62,051 | 124,193 | 0.96 | <0.001 | <0.001 | 77,943 | 93.0 | 4,087 | 4.9 | 1,824 | 2.2 |  |  |  |  |  |  |
|  | **Quadratic** | -61,670 | 123,465 | 0.95 | <0.001 | <0.001 | 77,768 | 92.7 | 4,189 | 5.0 | 1,898 | 2.3 |  |  |  |  |  |  |
|  | **Cubic** | -61,593 | 123,345 | 0.95 | <0.001 | <0.001 | 77,789 | 92.8 | 4,134 | 4.9 | 1,931 | 2.3 |  |  |  |  |  |  |
| **4-class** | **Linear** | -60,925 | 121,974 | 0.97 | <0.001 | <0.001 | 78,230 | 93.3 | 2,296 | 2.7 | 1,802 | 2.1 | 1,526 | 1.8 |  |  |  |  |
|  | **Quadratic** | -60,147 | 120,464 | 0.97 | <0.001 | <0.001 | 78,232 | 93.3 | 2,216 | 1.9 | 1,850 | 2.6 | 1,555 | 1.9 |  |  |  |  |
|  | **Cubic** | **-60,014** | **120,244** | **0.97** | **<0.001** | **<0.001** | **78,294** | **93.3** | **2,180** | **2.6** | **1,838** | **2.2** | **1,541** | **1.8** |  |  |  |  |
| **5-class** | **Linear** | -60,620 | 121,400 | 0.92 | <0.001 | <0.001 | 75,204 | 89.7 | 4,681 | 5.6 | 1,851 | 2.2 | 1,141 | 1.4 | 978 | 1.2 |  |  |
|  | **Quadratic** | -59,938 | 120,091 | 0.97 | <0.001 | <0.001 | 78,393 | 93.5 | 1,892 | 2.3 | 1,519 | 1.8 | 1,401 | 1.7 | 649 | 0.8 |  |  |
|  | **Cubic** | -59,782 | 119,836 | 0.97 | <0.001 | <0.001 | 78,147 | 93.2 | 2,214 | 2.6 | 1,472 | 1.8 | 1,198 | 1.4 | 823 | 1.0 |  |  |
| **6-class** | **Linear** | -60,433 | 121,059 | 0.94 | 0.007 | 0.008 | 76,063 | 90.7 | 3,505 | 4.2 | 1,754 | 2.1 | 1,309 | 1.6 | 613 | 0.7 | 611 | 0.7 |
|  | **Quadratic** | -59,745 | 119,751 | 0.96 | 0.010 | 0.009 | 77,903 | 92.9 | 1,609 | 1.9 | 1,543 | 1.8 | 1,123 | 1.3 | 927 | 1.1 | 748 | 0.9 |
|  | **Cubic** | -59,594 | 119,518 | 0.97 | <0.001 | <0.001 | 78,150 | 93.2 | 1,682 | 2.0 | 1,527 | 1.8 | 1,107 | 1.3 | 723 | 0.9 | 666 | 0.8 |

LL=log likelihood, BIC=Bayesian Information Criteria, VLMR=Vuong-Lo-Mendell-Rubin likelihood ratio test, BLRT= bootstrap likelihood ratio test; ^a^based on estimated posterior probabilities

**Figure S2.** Average trajectory group probabilities, grouped by most likely class membership: results derived from the 4-class latent class growth analysis model with cubic growth


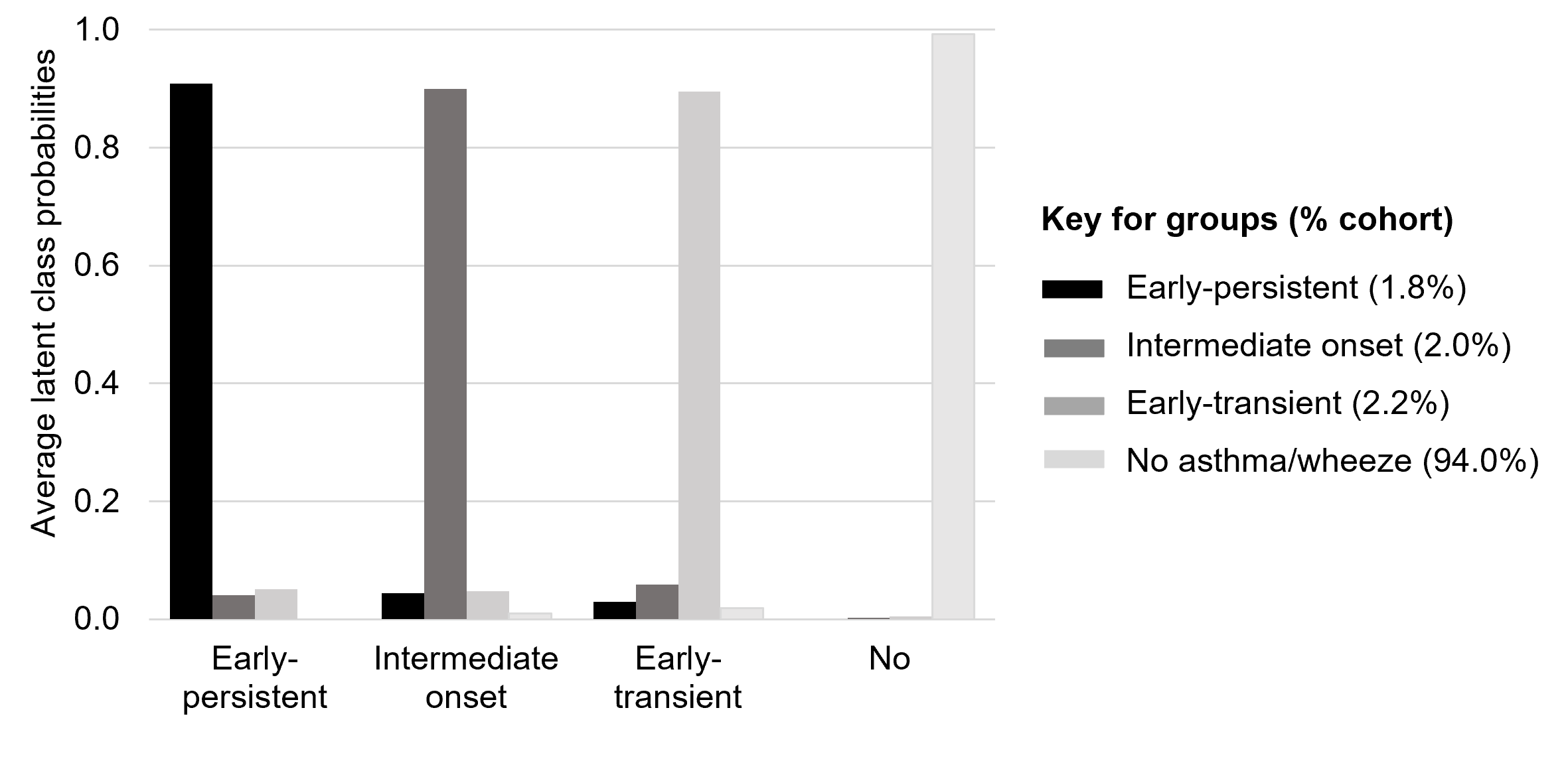


# References

1. NICE. Bronchiolitis in children: diagnosis and management. Published 2015. https://www.nice.org.uk/guidance/ng9/chapter/1-Recommendations#assessment-and-diagnosis

2. Ng MCW, How CH. Recurrent wheeze and cough in young children: is it asthma? *Singapore Med J*. 2014;55(5):236-241. doi:10.11622/smedj.2014064

3. Scott M, Kurukulaaratchy RJ, Arshad SH. Definitions are important and not all wheeze is asthma. *Thorax*. 2011;66(7):633. doi:10.1136/thx.2010.143941

4. Paediatric Formulary Committee. *BNF for Children 2012-2013*. Pharmaceutical Press; 2012.

5. Ferguson KD, McCann M, Katikireddi SV, et al. Evidence synthesis for constructing directed acyclic graphs (ESC-DAGs): a novel and systematic method for building directed acyclic graphs. *Int J Epidemiol*. 2019;(dyz150). doi:10.1093/ije/dyz150

6. Cole TJ, Williams AF, Wright CM. Revised birth centiles for weight, length and head circumference in the UK-WHO growth charts. *Ann Hum Biol*. 2011;38(1):7-11. doi:10.3109/03014460.2011.544139

7. Kristensen K, Hjuler T, Ravn H, Simões EAF, Stensballe LG. Chronic Diseases, Chromosomal Abnormalities, and Congenital Malformations as Risk Factors for Respiratory Syncytial Virus Hospitalization: A Population-Based Cohort Study. *Clin Infect Dis*. 2012;54(6):810-817. doi:10.1093/cid/cir928

8. National Records of Scotland. Vital Events - General Background Information. Published 2019. https://www.nrscotland.gov.uk/statistics-and-data/statistics/statistics-by-theme/vital-events/general-background-information

9. Owora AH, Zhang Y. Childhood wheeze trajectory-specific risk factors: a systematic review and meta-analysis. *Pediatr Allergy Immunol*. 2020;n/a(n/a). doi:10.1111/pai.13313

10. Jung T, Wickrama KAS. An Introduction to Latent Class Growth Analysis and Growth Mixture Modeling. *Soc Personal Psychol Compass*. 2008;2(1):302-317. doi:10.1111/j.1751-9004.2007.00054.x

11. Asparouhov T, Muthén B. Using Mplus TECH11 and TECH14 to test the number of latent classes. In: *Mplus Web Notes: No. 14*. ; 2012. https://www.tandfonline.com/doi/full/10.1080/10705510701575396

12. Nylund KL, Asparouhov T, Muthén B. Deciding on the number of classes in latent class analysis and growth mixture modeling: A Monte Carlo simulation study. *Structural Equation Modeling: A Multidisciplinary Journal*. 2007;14:535-569.

13. Nagin DS, Odgers CL. Group-Based Trajectory Modeling in Clinical Research. *Annu Rev Clin Psychol*. 2010;6(1):109-138. doi:10.1146/annurev.clinpsy.121208.131413

14. Wheeler AP, Worden RE, McLean SJ. Replicating Group-Based Trajectory Models of Crime at Micro-Places in Albany, NY. *J Quant Criminol*. 2016;32(4):589-612. doi:10.1007/s10940-015-9268-3

15. Feldman BJ, Masyn KE, Conger RD. New approaches to studying problem behaviors: a comparison of methods for modeling longitudinal, categorical adolescent drinking data. *Dev Psychol*. 2009;45(3):652-676. doi:10.1037/a0014851

16. Bellavia A, Zota AR, Valeri L, James-Todd T. Multiple mediators approach to study environmental chemicals as determinants of health disparities. *Environ Epidemiol*. 2018;2(2). https://journals.lww.com/environepidem/Fulltext/2018/06000/Multiple_mediators_approach_to_study_environmental.7.aspx

17. Hernán MA, Robins J. *Causal Inference: What If*. Chapman & Hall/CRC; 2020. https://www.hsph.harvard.edu/miguel-hernan/causal-inference-book/

18. Naimi AI, Schnitzer ME, Moodie EEM, Bodnar LM. Mediation Analysis for Health Disparities Research. *Am J Epidemiol*. 2016;184(4):315-324. doi:10.1093/aje/kwv329

19. Tchetgen Tchetgen EJ, VanderWeele TJ. On causal inference in the presence of interference. *Stat Methods Med Res*. 2012;21(1):55-75. doi:10.1177/0962280210386779

20. Rehkopf DH, Glymour MM, Osypuk TL. The Consistency Assumption for Causal Inference in Social Epidemiology: When a Rose Is Not a Rose. *Curr Epidemiol Rep*. 2016;3(1):63-71. doi:10.1007/s40471-016-0069-5

21. Scottish Intercollegiate Guidelines Network. *Bronchiolitis in Children: A National Clinical Guideline*.; 2006. http://resource.nlm.nih.gov/101300371

22. DEFRA. *Air Quality and Social Deprivation in the UK: An Environmental Inequalities Analysis*.; 2006. https://uk-air.defra.gov.uk/assets/documents/reports/cat09/0701110944_AQinequalitiesFNL_AEAT_0506.pdf

23. MacIntyre EA, Gehring U, Mölter A, et al. Air pollution and respiratory infections during early childhood: an analysis of 10 European birth cohorts within the ESCAPE Project. *Environ Health Perspect*. 2014;122(1):107-113. doi:10.1289/ehp.1306755

24. Lanari M, Prinelli F, Adorni F, et al. Risk factors for bronchiolitis hospitalization during the first year of life in a multicenter Italian birth cohort. *Ital J Pediatr*. 2015;41(1):40. doi:10.1186/s13052-015-0149-z

25. Moore HC, de Klerk N, Holt P, Richmond PC, Lehmann D. Hospitalisation for bronchiolitis in infants is more common after elective caesarean delivery. *Arch Dis Child*. Published online 2011. doi:10.1136/archdischild-2011-300607
